# Developments in the Study of Inert Gas Biological Effects and the Underlying Molecular Mechanisms

**DOI:** 10.3390/ijms26157551

**Published:** 2025-08-05

**Authors:** Mei-Ning Tong, Xia Li, Jie Cheng, Zheng-Lin Jiang

**Affiliations:** Institute of Special Environmental Medicine, Co-Innovation Center of Neuroregeneration, Nantong University, Nantong 226000, China; mn.tong@outlook.com (M.-N.T.); lixia7979@ntu.edu.cn (X.L.)

**Keywords:** inert gas, biological effect, molecular mechanism, research progress

## Abstract

It has long been accepted that breathing gases that are physiologically inert include helium (He), neon (Ne), nitrogen (N_2_), argon (Ar), krypton (Kr), xenon (Xe), and hydrogen (H_2_). The term “inert gas” has been used to describe them due to their unusually high chemical stability. However, as investigations have advanced, many have shown that inert gas can have specific biological impacts when exposed to high pressure or atmospheric pressure. Additionally, different inert gases have different effects on intracellular signal transduction, ion channels, and cell membrane receptors, which are linked to their anesthetic and cell protection effects in normal or pathological processes. Through a selective analysis of the representative literature, this study offers a concise overview of the state of research on the biological impacts of inert gas and their molecular mechanisms.

## 1. Introduction

Traditional inert gases—helium, neon, argon, krypton, xenon—alongside other low-reactivity diatomic gases, such as hydrogen and nitrogen, were historically utilized in early industrial gas encapsulation and deep-diving respiratory media due to their high biological safety and chemical stability. With rapid advances in molecular biology and structural pharmacology, the distinct regulatory roles of these gases in biological systems have become increasingly apparent. Nitrogen, once considered “biologically inert,” is now established to exert anesthetic effects. Xenon has emerged as a novel inhalation anesthetic, characterized by potent anesthetic and neuroprotective properties [1]. Similarly, hydrogen has demonstrated therapeutic potential in various disease models through specific antioxidant and anti-inflammatory mechanisms. Our understanding of the biological impacts of inert gas has evolved beyond the lipid-centric Meyer–Overton hypothesis, which emphasized lipid solubility, toward a framework focused on molecular interactions with protein targets [2]. This paradigm shift has not only redefined the theoretical foundations of gas pharmacology but also transformed inert gas—such as hydrogen—from mere “breathing media” or “anesthetics” into multi-target, multi-modal therapeutic gas molecules [3]. Instead of methodically incorporating all the pertinent literature, this paper integrates the biological effects, mechanisms, and application potential of inert gas through subjectively screening the representative literature. It is a narrative review that is based on both traditional research and state-of-the-art accomplishments in the field. Its goal is to refine research trends and provide directions for future exploration.

## 2. Physical and Chemical Properties of Inert Gas

Once considered physiologically inert, these gases are now recognized to demonstrate significant bioactivity as research progresses. The physiological responses of divers under hyperbaric conditions are critically dependent on the physicochemical properties of inert gas. The key physicochemical properties of these gases—including density, pressure, and solubility—are briefly summarized in Table 1 and discussed in the following section.

### 2.1. Solubility

Henry’s law states that the solubility of a gas in a liquid is directly proportional to its partial pressure [4]. Due to their inability to form hydrogen bonds or other strong intermolecular interactions, inert gas exhibits limited aqueous solubility, with van der Waals forces primarily governing their dissolution behavior [5]. Variations in inert gas solubility across different solvents reflect the specific characteristics and nature of solute–solvent interactions. Notably, inert gas demonstrates a substantially higher solubility—by several-fold—in lipids and oils than in water, a property termed lipid solubility. Among inert gas, xenon exhibits the greatest lipid solubility, exceeding that of helium by 100-fold. Consequently, other inert gas (helium, neon, argon, and krypton) displays lower lipid solubility than xenon. This elevated solubility in lipid-rich tissues underlies xenon’s superior anesthetic efficacy [6].

### 2.2. Pressure

All inert gases are colorless, odorless, and tasteless under standard temperature and pressure conditions. Nitrogen remains gaseous at room temperature under high pressure but liquefies when its critical pressure (3.39 MPa) is exceeded. Owing to its extremely low boiling point (−196 °C), liquid nitrogen is extensively employed in cryopreservation, cryosurgery, and cryogenic cooling applications. Helium, with its exceptionally low critical temperature (−268 °C), cannot undergo liquefaction at ambient temperature, even under extreme pressures. Given its low density and high thermal conductivity, helium is commonly utilized in diving gas mixtures, such as heliox. Neon similarly resists liquefaction at room temperature but sees limited application in diving medicine owing to its lower solubility and higher cost.

### 2.3. Density

The density of an inert gas increases with rising atomic number. Hydrogen is the lightest gas, followed by helium. Under standard conditions (0 °C, 1 atm), nitrogen exhibits a density of 1.2506 g/L. The gas density critically influences the respiratory function and is therefore a key parameter for selecting inert gas in diving gas mixtures. With a density of 0.1786 g/L, helium is particularly suitable for deep diving due to its ability to substantially reduce breathing resistance. Neon, argon, krypton, and xenon display densities of 0.9002 g/L, 1.7840 g/L, 3.7490 g/L, and 5.8870 g/L, respectively [7]. High-density gases are generally avoided in diving medicine, as they increase the breathing effort and may exhibit potent anesthetic effects.

### 2.4. Other Aspects

The melting and boiling points of inert gas increase with rising atomic number. Helium exhibits a melting point of −272.2 °C, compared with −111.79 °C for xenon [8]. Similarly, their boiling points measure −268.9 °C and −108.12 °C, respectively. The transport properties, diffusion rate, isobaric specific heat capacity, and thermal conductivity at 0 °C are inversely correlated with the atomic number. These relationships are systematically summarized in Table 1.

## 3. Biological Effects of Inert Gas

Inert gas exerts diverse biological effects in living organisms, encompassing anesthetic actions, organ-protective properties, and cellular function regulation. These effects demonstrate significant physiological relevance across multiple pathological and physiological states.

### 3.1. Biological Effects of Helium

Helium occupies the highest position in the inert gas group (Group 18) of the periodic table. Unlike xenon, which exhibits anesthetic properties under standard clinical pressures, helium requires extreme hyperbaric conditions (approximately 189 atm) to produce anesthetic effects. This pressure threshold induces high-pressure neurological syndrome (HPNS), introducing confounding physiological variables that render helium impractical for perioperative anesthesia [7]. Contrary to initial expectations, experimental studies utilizing both preconditioning and postconditioning methodologies have demonstrated helium’s organ-protective properties against ischemia–reperfusion pathology in cardiac and neurological systems [1,9,10].

### 3.2. Biological Effects of Hydrogen

As an emerging inert gas, hydrogen demonstrates protective effects in multiple organ injury models under atmospheric pressure. For example, studies confirmed its neuroprotective properties [11], mitigation of adverse outcomes in myocardial ischemia–reperfusion injury [12], and therapeutic potential in renal diseases through improved renal function and reversal of pathological changes [13]. Although hydrogen exhibits greater anesthetic potency than helium, its small molecular size reduces respiratory resistance during deep diving. Early research established the safety and feasibility of inhaling 97% H_2_/3% O_2_ mixtures at 10 atmospheres [14]. Following this discovery, the Royal Swedish Navy implemented hydrogen in deep-sea diving operations from 1943 onward to prevent decompression sickness—a practice continuing to date [15]. Despite combustion risks, hydrogen’s combination of half of helium’s molecular mass and moderate anesthetic potency suggests potential for replacing conventional diving gases when properly managed [16]. Clinical investigations of hydrogen mixtures in deep-sea saturation diving reveal effectiveness in alleviating HPNS without significant adverse effects [17].

### 3.3. Biological Effects of Nitrogen

Nitrogen is extensively utilized in medical and food preservation applications—including powering surgical instruments and extending food shelf life—due to its chemical inertness at atmospheric pressure. However, during deep diving, inhalation of compressed air impairs diver consciousness and physiological function under high-pressure conditions. This manifests through multiple symptoms: sensory dulling, impaired motor coordination, cognitive decline, hallucinations, and eventual unconsciousness. Comprehensive investigation has established nitrogen as the primary etiological agent of these manifestations [18,19], a phenomenon termed nitrogen narcosis. The anesthetic effect initiates at approximately 30 m in depth (0.3 MPa; 4 ATA), progressively intensifying with increasing pressure. Complete unconsciousness may occur around 100 m in depth [20]. Notably, interspecies variation exists, with rats requiring ~1 MPa pressure to elicit comparable narcotic effects [21].

### 3.4. Biological Effects of Argon

Argon, the most abundant inert gas in the atmosphere (~9340 ppm) [22], exhibits significantly stronger anesthetic potency than nitrogen. When divers inhale argon-containing mixtures under hyperbaric conditions, characteristic impairments emerge: delayed cognition, reduced manual dexterity, mood alterations, and potential unconsciousness [19]. Beyond its anesthetic properties, argon demonstrates notable neuroprotective effects across animal models—including traumatic brain injury (TBI) and transient middle cerebral artery occlusion (tMCAO)—under normobaric conditions [1]. Post-treatment with argon following retinal ischemia–reperfusion injury protects ganglion cells through dose- and time-dependent suppression of pro-apoptotic proteins [23]. Argon’s therapeutic significance extends beyond neuroprotection. In a porcine model of ectopic autologous renal transplantation, argon-saturated preservation solution significantly enhanced renal functional recovery, prolonged graft survival, ameliorated tissue damage, and activated antioxidant defenses [24]. Complementary in vitro and in vivo studies further confirm argon’s cardioprotective properties [9].

### 3.5. Biological Effects of Xenon

Xenon, among the earliest inert gases comprehensively studied, had its anesthetic properties first demonstrated by Lawrence et al. in 1946 [25]. Subsequent research established its superior hemodynamic stability and favorable safety profile in clinical applications [26]. Recent studies indicate xenon anesthesia correlates with a reduced frontal electroencephalogram peak alpha frequency [27]. Beyond anesthetic properties, xenon exerts significant neuroprotective effects, which was established across multiple models; including TBI; neonatal hypoxic–ischemic encephalopathy (HIE); and neurodegenerative disorders, such as Parkinson’s and Alzheimer’s diseases [1,28].

### 3.6. Potential Adverse Effects of Inert Gas

Even though inert gas has well-established positive biological effects, such as neuroprotective qualities and anesthetic uses, this research emphasizes how important it is to assess any potential negative physiological effects. Interestingly, exposure to xenon has been shown to cause severe erythrocyte damage, which is characterized by noticeable hemolysis and noticeable changes in the shape of red blood cells [29]. Due to its high diffusivity and limited blood solubility, helium poses special hazards when used alone. These physical characteristics greatly raise the chance of dangerous intravascular gas embolism development, a major complication with potentially dire clinical repercussions [30]. These results demonstrate the need for a thorough safety evaluation and context-specific application since the therapeutic potential of inert gas must be carefully weighed against their unique physicochemical concerns.

## 4. Mechanisms of Generation of Biological Effects of Inert Gas

### 4.1. Lipid-Related Theory

The Meyer–Overton lipid theory, formulated based on experimental observations, establishes a positive correlation between anesthetic potency and lipid solubility: anesthetic efficacy increases proportionally with lipophilicity. Also known as the Meyer–Overton rule [31], this principle posits that anesthetic potency depends on a substance’s capacity to penetrate neuronal membrane lipid bilayers and modify their physical properties. Nitrogen exemplifies this relationship, exhibiting strong anesthetic effects at specific partial pressures due to its high lipid solubility [20]. To quantify relative narcotic potency, nitrogen anesthesia is conventionally assigned a reference value of 1 (Table 2) [6]. The anesthetic potentials of other inert gases are then derived through comparative assessment against this nitrogen benchmark.

Although anesthetic action has historically targeted cell membranes, the mechanistic link between membrane effects and ion channel modulation remains incompletely elucidated. Inhalational anesthetics, such as chloroform and isoflurane, disrupt phospholipase D2 localization within lipid rafts, thereby impacting the enzyme’s production of phosphatidic acid signaling lipids. This cascade ultimately activates the TWIK-related potassium channel (TREK-1), producing anesthetic effects—findings that substantiate a membrane-mediated anesthesia mechanism [32]. Lipid rafts constitute dynamic microdomains on cell membranes that are enriched with specific proteins, cholesterol, and phospholipids. As critical regulators of membrane protein localization and function, they facilitate rapid assembly/dissociation, participate in cellular signaling, maintain membrane stability and fluidity, and mediate material transport [33]. Molecular dynamics simulations reveal that at concentrations of two xenon atoms per lipid molecule, xenon spontaneously coalesces into nanobubbles within lipid raft bilayers, rapidly embedding while displacing cholesterol molecules essential for membrane stability. Increasing the xenon concentration to three atoms per lipid molecule substantially enlarges the nanobubbles, ultimately compromising the bilayer integrity [34]. However, research progression has revealed limitations in the lipid theory’s capacity to account for experimental observations. Clinically relevant anesthetic concentrations produce no detectable alterations in lipid bilayers [35]. Moreover, the minimum alveolar concentration paradoxically increases with temperature elevation—contradicting lipid theory predictions [36,37]. These findings collectively indicate that the lipid theory inadequately explains anesthetic mechanisms, necessitating alternative conceptual frameworks.

### 4.2. Protein Theory

**Figure 1:**
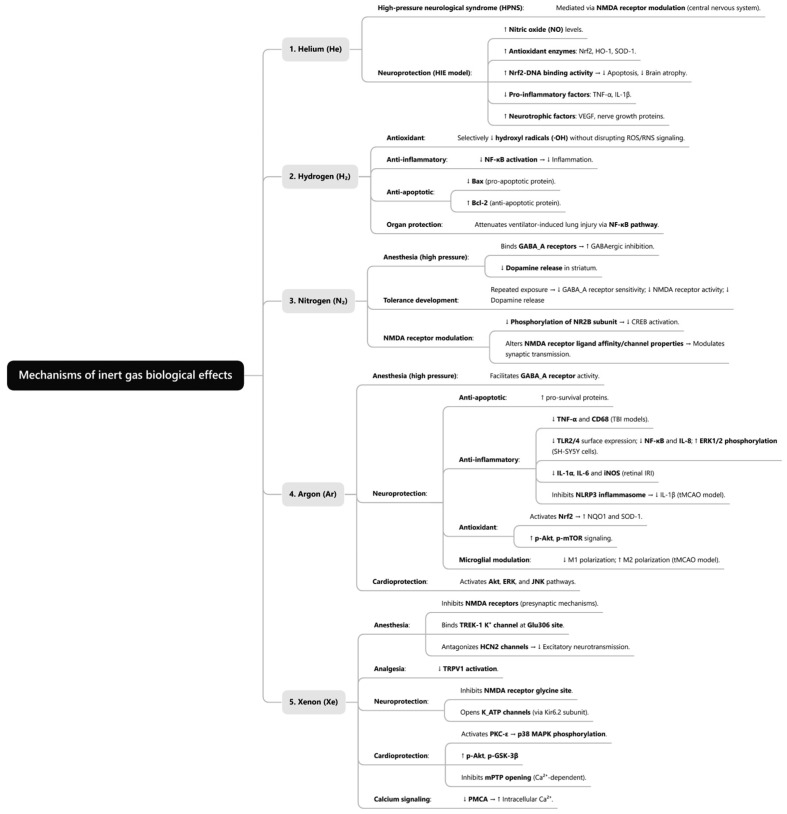
Mechanism of biological effects of inert gases.

A seminal 1984 study by Franks and Lieb demonstrated that anesthetic concentrations of halothane could inhibit the activity of purified soluble proteins (e.g., firefly luciferase) by up to 50% [38]. This pivotal finding redirected investigations toward protein targets, suggesting anesthetic effects arise through direct molecular binding to proteins. Consequently, research shifted from the lipid hypothesis toward protein-centered mechanisms of anesthesia. Inert gas modulates diverse protein targets—including ion channels, pumps, receptors, and inflammatory mediators—thereby influencing physiological processes through protein interactions [39]. These observations indicate that inert gases exert their distinct pharmacological effects by regulating biological processes, such as intracellular signaling, ion homeostasis, and inflammatory responses via protein binding. Crucially, such biological outcomes remain intrinsically linked to each gas’s physicochemical properties. The following sections systematically examine biological mechanisms of extensively studied inert gas (Figure 1).

#### 4.2.1. Helium

Helium–oxygen mixtures are commonly employed as breathing media in deep-sea saturation diving. However, divers exceeding 150 m in depth risk developing HPNS, a condition primarily attributable to hyperbaric helium exposure rather than elevated pressure alone [40,41]. Recent evidence indicates that HPNS involves helium-mediated modulation of central nervous system N-methyl-D-aspartate (NMDA) receptors [42]. Notably, pretreatment with 70% He/30% O_2_ in HIE models demonstrates neuroprotective efficacy through multiple mechanisms: significant elevation of nitric oxide; reduced cerebral infarct volume; and enhanced antioxidant defenses, including nuclear factor erythroid 2-related factor 2 (Nrf2), heme oxygenase-1 (HO-1), and superoxide dismutase-1 (SOD-1) activity with a concomitant increase in Nrf2-DNA binding. These changes attenuate apoptosis, improve neurological function, and reduce brain atrophy [43]. Additionally, such pretreatment stimulates vascular endothelial growth factor (VEGF) biosynthesis and neurotrophic protein expression while significantly suppressing the pro-inflammatory mediators tumor necrosis factor-α (TNF-α) and interleukin-1β (IL-1β) in cerebral tissue, collectively enhancing neurobehavioral outcomes [44].

#### 4.2.2. Hydrogen

The biological effects of hydrogen under atmospheric pressure primarily involve anti-inflammatory, anti-apoptotic, and antioxidant properties. Mechanistic studies reveal that hydrogen diffuses into cells and organelles (e.g., mitochondria, nuclei), selectively reducing hydroxyl radical levels without disrupting other reactive oxygen species (ROS)/reactive nitrogen species (RNS) or metabolic redox signaling. This selective antioxidant action ensures hydrogen’s safety profile [45]. Hydrogen modulates inflammation through direct regulation of intracellular oxidative status and nuclear factor-κB (NF-κB) activity [13]. Additionally, hydrogen treatment downregulates pro-apoptotic Bcl-2-associated X protein (Bax)expression while upregulating anti-apoptotic B-cell lymphoma 2 (Bcl-2) protein, demonstrating significant anti-apoptotic efficacy. Through this NF-κB pathway, hydrogen effectively mitigates inflammation and apoptosis in ventilator-induced lung injury models [46].

#### 4.2.3. Nitrogen

Albert Behnke first postulated in 1935 that elevated nitrogen partial pressure underlies diving-associated anesthesia [47]. Mechanistic investigations reveal that gabapentin—a competitive γ-aminobutyric acid type A (GABA_A_) receptor antagonist—significantly increases the pressure threshold for nitrogen narcosis, indicating nitrogen may selectively potentiate GABA_A_ receptor function to exert anesthetic effects [48]. High-pressure nitrogen exposure substantially reduces striatal dopamine release while enhancing GABA_A_ receptor activation. Repeated nitrogen anesthesia induces persistent dopamine depletion and suppresses NMDA receptor activity, concurrently promoting GABA_A_ receptor desensitization and tolerance development [21]. Recent studies demonstrated that nitrogen reduces phosphorylation of the NMDA receptor subunit N-methyl-D-aspartate receptor subtype 2B (NR2B), consequently inhibiting activation of the downstream cAMP-response element binding protein (CREB) [49]. These findings establish nitrogen’s capacity to modulate NMDA receptor channel properties and ligand affinity, thereby altering synaptic transmission and neuronal excitability.

#### 4.2.4. Argon

Argon efficiently penetrates biological tissues—including deep central nervous system structures—due to its non-polar nature and high diffusivity. This property enables access to internal domains of target proteins. Through molecular interactions with proteins, argon initiates cascading cellular signaling alterations that ultimately mediate diverse biological effects [1].

Under hyperbaric conditions, argon directly modulates GABA_A_ receptors to produce anesthesia. Both flumazenil (a benzodiazepine-site antagonist) and gabapentin (a competitive GABA_A_ receptor antagonist) significantly elevate the pressure threshold for argon-induced abolition of the righting reflex. This demonstrates that argon’s anesthetic mechanism involves GABA_A_ receptor facilitation, with antagonists exerting effects through direct interference with receptor activation [48].

Argon’s neuroprotective effects at atmospheric pressure are primarily mediated through inhibition of apoptosis, oxidative stress, and inflammatory responses. In experimental TBI models, argon inhalation demonstrated significant neuroprotection, attenuating inflammatory responses and enhancing the antioxidant capacity. Treated subjects exhibited accelerated functional recovery, improved neurological scores, and reduced histopathological damage [50]. Mechanistically, argon administration suppressed TBI-induced expression of the proinflammatory markers TNF-α and CD68 while upregulating phosphorylated protein kinase B (p-Akt) signaling and activating the cytoprotective transcription factor Nrf2. In human neuroblastoma SH-SY5Y cells, argon preconditioning dose-dependently attenuated rotenone-induced apoptosis [51], with 2 h 74% argon exposure decreasing the Toll-like receptor 2/4 (TLR2/4) surface expression, enhancing extracellular regulated protein kinase (ERK1/2) phosphorylation while reducing NF-κB and Akt activation, suppressing mitochondrial apoptosis and the heat shock response, and inhibiting interleukin-8 expression. In rat models of retinal ischemia/reperfusion injury (IRI), argon treatment attenuates microglial activation and suppresses inflammatory responses, concomitantly reducing expression levels of interleukin-1α (IL-1α), IL-1β, interleukin-6 (IL-6), TNF-α, and inducible nitric oxide synthase (iNOS) [52]. Furthermore, in the tMCAO model, argon inhibited NLRP3 inflammasome activation and IL-1β release through NF-κB pathway suppression—which modulates degradation of proteins like gasdermin D (GSDMD)—while promoting post-ischemic M2-type polarization and suppressing microglial activity with a concurrent reduction in M1-type polarization [53]. Research in neonatal HIE models reveals a novel molecular basis for argon’s neuroprotective potential. Cortical neuronal cultures exposed to argon demonstrated attenuation of oxidative stress, neuroinflammation, and neuronal apoptosis alongside reduced cerebral infarction volume. This treatment concurrently upregulated phosphorylated mTOR (p-mTOR) and activated the transcription factor Nrf2, conferring cytoprotection against oxygen–glucose deprivation. In vivo, argon exposure substantially enhanced Nrf2 signaling, elevating downstream effectors NAD(P)H: quinone oxidoreductase 1 (NQO1) and SOD-1 [54]. Moreover, argon provides neuroprotection against moderate-to-severe hypoxic–ischemic brain injury, potentially mediated through increased synthesis of pro-survival proteins such as Bcl-2 [55]. Pretreatment with argon also protects human cardiac myocyte-like progenitor cells from apoptosis during ischemic conditions via Akt and ERK activation with biphasic c-Jun N-terminal kinase (JNK) regulation [56]. Given its inherent stability, cost-effectiveness, and practical administration, these preclinical findings position argon as a promising therapeutic candidate for tissue protection and ischemia–reperfusion injury mitigation.

#### 4.2.5. Xenon

At atmospheric pressure, xenon exhibits potent anesthetic properties distinct from other inert gases. Mechanistic studies demonstrate that xenon anesthesia arises through specific modulation of glutamatergic signaling pathways. Electrophysiological investigations by Nonaka et al. established that presynaptic reduction of excitatory transmission constitutes the primary anesthetic mechanism, with minimal GABAergic synapse involvement [57]. Subsequent research confirmed that xenon predominantly inhibits NMDA receptor responses via presynaptic mechanisms [58]. Furthermore, TREK-1—a two-pore domain (K2P) potassium channel—was identified as a selective molecular target for xenon-induced general anesthesia, with glutamic acid 306 (Glu306) likely representing the critical binding site for anesthetic effects [59]. Recent in silico neuronal simulations substantiate xenon’s narcotic efficacy through antagonism of hyperpolarization-activated cyclic nucleotide-gated channel 2 (HCN2) and suppression of glutamatergic neurotransmission [27]. Emerging evidence suggests xenon’s analgesic properties may partially arise from attenuated signaling through transient receptor potential vanilloid1 (TRPV1), a key mediator in specific inflammatory nociceptive pathways [60]. Harris et al. propose that xenon’s neuroprotective effects against traumatic brain injury involve NMDA receptor glycine site inhibition [61]. Bantel et al. identified xenon as a novel adenosine triphosphate-sensitive potassium (K_ATP_) channel opener that directly targets the Kir6.2 pore-forming subunit, reducing ATP-mediated channel inhibition and enhancing K_ATP_ currents—indicating neuroprotective potential [62]. In rat hearts, Weber et al. demonstrated xenon-induced cardioprotection via pharmacological preconditioning, with protein kinase C epsilon (PKC-ε) activation and subsequent p38 mitogen-activated protein kinase (p38 MAPK) phosphorylation constituting central mechanisms [63]. Mio et al. reported that xenon preconditioning reduces myocardial infarction size through Akt and glycogen synthase kinase 3 beta (GSK-3β) phosphorylation, mitochondrial preservation, and inhibition of calcium-induced mitochondrial permeability transition pore (mPTP) opening [64]. Additionally, xenon inhibits the activity of the plasma membrane Ca^2+^-ATPase (PMCA), resulting in a decrease in the transport capacity of calcium ions within C6 glioma cells, so the concentration of free calcium ions in the cells increases [65]. Collectively, xenon modulates multiple cellular targets—including ion channels, receptors, and signaling pathways—mediating both anesthesia and organ protection effects.

## 5. Challenges in Translating Laboratory Discoveries to Clinical Practice

Taking inert gas treatments from the lab to real patients faces several practical hurdles. First, doctors need specialized breathing equipment, like closed-circuit systems, to deliver gases such as xenon safely, making the process more complex. Second, finding effective doses is challenging—for example, brain-protecting argon levels far exceed natural air concentrations, requiring expensive hospital machines. Finally, cost and supply issues create barriers: xenon’s rarity makes it extremely expensive, hindering large human studies, while helium access can be disrupted by global politics, causing shortages.

## 6. Summary and Outlook

Research on the biological effects of inert gas has advanced substantially, revealing multi-target and multi-pathway mechanisms underlying their anesthetic, anti-inflammatory, antioxidant, and cytoprotective properties. These findings not only provide molecular evidence supporting clinical applications but also demonstrate therapeutic potential in organ protection, metabolic disorders, and neurological conditions. Future investigations should integrate cryo-electron microscopy, molecular dynamics simulations, and multi-omics approaches to examine synergistic inert gas–lipid–protein interactions and evaluate gas–protein binding dynamics. Furthermore, comprehensive clinical trials are required to assess the therapeutic safety and efficacy across pathologies; investigate combination strategies with conventional therapies to enhance outcomes and reduce adverse effects; explore novel applications, including immunomodulation and oncology; and develop optimized delivery systems meeting translational and clinical demands. Through interdisciplinary collaboration and technological innovation, inert gases are poised to transition from fundamental research to clinical application.

## Figures and Tables

**Table 1:** The physicochemical characteristics of inert gas.

Gas	Molecular Weight	Diffusion Coefficient	Solubility in Lipid	Oil–Water Solubility Ratio	Density at STP *	Specific Heat Capacity at Constant Pressure	Thermal Conductivity at 0 °C
H_2_	2	0.41	0.036	2.1	0.0899	14.31	0.180
He	4	0.35	0.015	1.7	0.1785	5.19	0.144
Ne	20	0.32	0.019	2.07	0.9002	1.03	0.049
N_2_	28	0.20	0.067	5.2	1.2506	1.04	0.024
Ar	40	0.16	0.14	5.3	1.7804	0.52	0.017
Kr	83.7	0.11	0.43	9.6	3.7360	0.25	0.009
Xe	131.3	0.08	1.7	20.0	5.8870	0.16	0.005

* STP refers to standard temperature and pressure.

**Table 2:** Correlation of the narcotic potency of inert gases with liposolubility.

Gas	Molecular Weight	Solubility in Lipid	Oil–Water Solubility Ratio	Relative Narcotic Potency
He	4	0.015	1.7	0.23
Ne	20	0.019	2.07	0.28
H_2_	2	0.036	2.1	0.55
N_2_	28	0.067	5.2	1.00
Ar	40	0.14	5.3	2.33
Kr	83.7	0.43	9.6	7.14
Xe	131.3	1.7	20.0	25.64

## Data Availability

No new data were created in this study. Data sharing is not applicable to this article.
